# Current Pharmacologic Approaches for Prevention and Treatment of Bronchopulmonary Dysplasia

**DOI:** 10.1155/2012/598606

**Published:** 2012-01-03

**Authors:** Kristen Tropea, Helen Christou

**Affiliations:** ^1^Division of Newborn Medicine, Children's Hospital Boston and Harvard Medical School, Boston, MA 02115, USA; ^2^Division of Newborn Medicine, Brigham and Women's Hospital and Harvard Medical School, Boston, MA 02115, USA

## Abstract

Bronchopulmonary dysplasia (BPD) is a major complication of preterm birth and has serious adverse long-term health consequences. The etiology of BPD is complex, multifactorial, and incompletely understood. Contributing factors include ventilator-induced lung injury, exposure to toxic oxygen levels, and infection. Several preventive and therapeutic strategies have been developed with variable success. These include lung protective ventilator strategies and pharmacological and nutritional interventions. These strategies target different components and stages of the disease process and they are commonly used in combination. The purpose of this review is to discuss the evidence for current pharmacological interventions and identify future therapeutic modalities that appear promising in the prevention and management of BPD. Continued improved understanding of BPD pathogenesis leads to opportunities for newer preventive approaches. These will need to be evaluated in the setting of current clinical practice in order to assess their efficacy.

## 1. Introduction

Bronchopulmonary dysplasia (BPD) remains a major complication of prematurity resulting in significant mortality and morbidity despite advances in perinatal care and decline in mortality rates among very low birth weight (VLBW) infants [1]. Increased survival among VLBW infants contributes to the overall increase in the incidence of BPD and currently infants with birth weights <1250 grams account for 97% of cases of BPD [2]. The long-term health consequences of BPD include respiratory disease that can persist into adulthood and increased susceptibility to respiratory infections, asthma, pulmonary hypertension, repeated hospitalizations, neurodevelopmental impairment, and also increased mortality [3]. The etiology of BPD is multifactorial and includes exposure to mechanical ventilation, oxygen toxicity, infection, and inflammation that contribute to arrested alveolar development and associated abnormal vascular growth and damage to the distal airways of the highly vulnerable premature lung [4]. Multiple pharmacological and nonpharmacological approaches have been proposed for the prevention or treatment of preterm lung injury and BPD. While antenatal steroids, protective ventilation strategies, targeted oxygen saturation goals, caffeine therapy, vitamin A therapy, and optimization of nutrition have helped to modestly improve BPD outcomes, most current therapies are supportive [4, 5]. Many therapies remain controversial due to unacceptable side effects and others continue to need further study including randomized controlled trial testing and long-term outcome follow-up analysis.

In this review, we present the current and potential future pharmacologic approaches for the prevention and management of BPD based on published meta-analyses, randomized controlled trials, systematic reviews, individual clinical studies, and emerging work from animal models of disease. A comprehensive list of the drugs discussed in the prevention and management of BPD is shown in Table 1.

## 2. Caffeine

Caffeine is a methylxanthine commonly used in the treatment of apnea of prematurity, a common complication of prematurity occurring in at least 85 percent of infants who are born at less than 34-week gestation [6, 7]. Methylxanthines have been shown to reduce the frequency of apnea of prematurity and need for mechanical ventilation during the first seven days of therapy [8]. A recent large randomized controlled trial followed primary outcome of long-term neurodevelopment and secondary short-term outcomes of rates of BPD in infants with birthweights from 500 to 1250 grams [8]. Infants in the caffeine group were found to have an incidence of BPD of 36% compared to 47% of infants in the placebo group [8]. Patients in the caffeine group had reduced weight gain, but this was found to be temporary. Long-term follow-up at 18 to 22 months showed that infants assigned to the caffeine group had a lower incidence of neurodevelopmental impairment including lower rates of cerebral palsy and lower rates of cognitive delay [9]. The potential mechanism of the effect of caffeine on decreased incidence of BPD remains unknown. The number of days on positive pressure ventilation was decreased by one week in the infants assigned to the caffeine group which could account for potential reduction in ventilator-induced lung injury in the caffeine group [8, 9]. When interpreting this secondary outcome of caffeine leading to a decreased rate of BPD, it is important to take into consideration that the randomization protocol effectively excluded infants who needed mechanical ventilation for greater than 10 days, in other words infants at a presumably higher risk for BPD. In conclusion, the evidence supports the use of caffeine in treatment of apnea of prematurity with findings of secondary benefits including reduction in BPD rates and improved neurodevelopmental outcomes.

## 3. Diuretics

Diuretics are a common class of drugs used in the management of BPD. Interstitial alveolar edema appears to be a prominent feature of BPD and excessive interstitial edema can lead to decreased lung compliance. Iatrogenic increase in fluid administration, capillary leak from inflammation due to infection or from ventilator-induced lung injury, and volume overload due to left to right shunting through a patent ductus arteriosus are some of the factors that contribute to pulmonary edema [11–13]. Diuretics potentially benefit by increasing reabsorption of fluid from the lung.

**(a) Loop Diuretics**

Loop diuretics act by blocking the luminal Na-K-2Cl transporter in the thick ascending limb of the loop of Henle. Loop diuretics compete for the chloride site on this transporter and diminish reabsorption. Furosemide is the most widely administered loop diuretic in neonates; yet its use remains controversial. A Cochrane meta-analysis has reviewed several small trials that studied the risks and benefits of systemic furosemide on preterm infants with BPD [14]. Minimal effect was observed with enteral furosemide in preterm infants <3 weeks of age [14]. Chronic administration of furosemide for a week improved short-term outcomes of pulmonary compliance, oxygen requirement, and minute ventilation in preterm infants >3 weeks of age with BPD [15–17]. However, no existing trials have addressed long-term outcomes including effect on duration of oxygen requirement, weaning off mechanical ventilation, duration of hospital stay, incidence of BPD, and mortality [14].

Administration of aerosolized furosemide has also been explored in an effort to minimize systemic side effects in preterm infants with evolving BPD. A Cochrane meta-analysis reviewed 8 trials with aerosolized furosemide and noted that a single-aerosolized dose of furosemide may transiently improve pulmonary mechanics in preterm infants >3 weeks of age [18]. There was no significant pulmonary improvement with chronic administration of aerosolized furosemide. None of these studies examined delivery of the drug to the distal airways or serum levels. Furthermore, the studies had inadequate assessment of clinical outcomes such as duration of mechanical ventilation, oxygen requirement, length of stay, incidence of BPD, mortality, and complications of treatment.

In summary, the data on the use of furosemide is limited. Potential risks of loop diuretic therapy such as electrolyte imbalance, ototoxicity, nephrocalcinosis, and osteopenia along with inconclusive data on long-term primary and secondary outcomes warrant future trials to justify the chronic use of furosemide in current clinical practice. Current evidence does not support use of loop diuretics for prevention of BPD and use of furosemide sparingly to acutely treat pulmonary edema is currently the preferred practice.

**(b) Thiazides**

Thiazides effect the early portion of the distal tubule and bind directly to the chloride site of the electroneutral sodium chloride channel. The risk of electrolyte abnormalities is far less with thiazide diuretics compared to loop diuretics due to the small amount of sodium absorption occurring in the distal tubule. A Cochrane meta-analysis examined six studies on the use of thiazides in preterm infants and found that chronic use of thiazides improves lung mechanics and decreases the need for supplemental furosemide boluses [19]. In a randomized double-blind placebo-controlled trial thiazide and spironolactone were given to 43 nonintubated BPD patients until they no longer required oxygen supplementation [20]. The study showed decreased oxygen requirement and improved lung function in the treatment group compared to placebo but failed to show any improvement in the survival rate, duration of oxygen requirement, or length of hospital stay. In intubated patients, a lower oxygen requirement and better lung compliance and decreased administration of furosemide boluses in the treatment group compared to placebo were found in one study, but with no change in airway resistance [21]. Administration of thiazides did not decrease the length of hospital stay, need for ventilator support, or other long-term outcomes. Addition of potassium-sparing diuretics such as spironolactone which act exclusively on the Na−K/H exchange mechanisms in the late distal tubule and cortical collecting duct did not alter the compliance or oxygen requirement compared to thiazides alone and had no effect on need for electrolyte supplementations [22].

Further studies on the role of chronic diuretics in the treatment of BPD may be warranted in the current practice era of antenatal steroids and surfactant therapy in order to provide definitive evidence of their clinical usefulness. No clear evidence is present for use of thiazide diuretics for the prevention or management of BPD. Meanwhile thiazides are the diuretics of choice in ventilator-dependent infants for specific case-based administration and care should be taken to avoid electrolyte abnormalities with appropriate supplementation.

## 4. Bronchodilators

BPD causes increased airway resistance due to smooth muscle hypertrophy and hyperreactivity [3]. Bronchodilators are a common medication used to relieve bronchospasm in asthmatic patients and have been studied in BPD patients. Studies have shown that bronchospasm contributes to elevated pulmonary resistance in preterm infants and bronchodilators improve dynamic compliance by lowering pulmonary resistance [23–26]. Bronchodilators have been broadly categorized into adrenergic and anticholinergic agents. Their effect is transient and both have been shown to acutely reduce pulmonary resistance and increase compliance in BPD patients. Variability in individual responsiveness to *β*-agonists may be genetically determined [27–29]. In the Cochrane database only one trial addressed the use of bronchodilators for the prevention of BPD and measured long-term outcomes [30]. The study enrolled 173 infants <31 weeks of gestational age, who needed ventilatory support at the 10th postnatal day. They were randomized to four groups and received either placebo, placebo with salbutamol or beclomethasone, or both beclomethasone and salbutamol for 28 days. No significant effects of the treatment on incidence or severity of BPD, duration of ventilator support, or oxygen therapy were observed.

The two most widely used bronchodilators are albuterol and ipratropium [23, 31]. Potential side effects of *β* sympathomimetic agents include tachycardia, hypokalemia, arrhythmias, and hyperglycemia. Inhaled anticholinergic agents, in addition, decrease gastrointestinal motility and dry and thicken respiratory secretions. Ipratropium has traditionally been used along with albuterol to provide synergism. No trials have yet investigated if a combination therapy of a beta agonist and anticholinergic result in improved outcomes in BPD compared to albuterol alone. Future trials are required to study various modes of delivery of the different adrenergic and anticholinergic drugs alone or in combination.

Due to a low number of trials and potential side effects, current evidence supports that bronchodilator therapy should be limited to infants with evidence of bronchospasm and continued only if there is a clinical response to therapy. Even in these cases, there is no evidence that the long-term outcome is altered.

## 5. Steroids 

Inflammation is a main contributor to the pathogenesis of BPD. Since corticosteroids are potent anti-inflammatory agents, many trials have examined the use of steroids in BPD. Systemic steroid administration reduces the inflammatory response, produces a rapid improvement in pulmonary function with better gas exchange, and facilitates weaning from mechanical ventilation. In addition to the anti-inflammatory effects, steroids also enhance surfactant production, decrease airway edema, stabilize capillary leakage, augment *β* adrenergic activity, and decrease overall lung fibrosis [32–34]. Both systemic and inhaled corticosteroids have been studied extensively in preterm neonates for prevention and treatment of BPD. The steroid trials may be categorized according to the time of administration. Early administration is defined as less than eight days after birth. A Cochrane meta-analysis reviewed that twenty-eight randomized controlled trials evaluated effects of early treatment of dexamethasone on the incidence of BPD [35, 36]. Steroids facilitated extubation and decreased the incidence of BPD. However, adverse effects such as hyperglycemia, gastrointestinal perforation, hypertension, infection, steroid-induced cardiomyopathy, and long-term neurodevelopmental effects including cerebral palsy complicated the treatment. Moderately early administration of dexamethasone (between 7 to 14 days) led to similar decrease in the incidence of BPD and facilitated extubation [37]. Nine trials studied late administration of dexamethasone usually after 3 weeks [38]. These studies showed transient improvement including increased success rates of extubation and reduction of the need for later steroid and home oxygen therapy compared to the controls. Both moderately early and late treatments were complicated by short- and long-term side effects. The most worrisome long-term effect increased risk for poor neurological outcome including cerebral palsy. As a result, the European Association of Perinatal Medicine, the American Academy of Pediatrics, and the Canadian Pediatric Society have advised against routine use of systemic dexamethasone for the prevention or treatment of BPD. One study has examined the use of low-dose dexamethasone (0.89 mg/kg over 10 days) in preterm infants who were ventilator dependent after 1 week of age [39]. The study showed decreased ventilator requirement, improved oxygenation, and greater percentage of successful extubation in the treatment group compared to placebo. Although the study showed no short-term side effects such as hypertension or intestinal perforation, it enrolled relatively “older” premature infants (mean gestational age 28-29 weeks) and no long-term outcomes were included.

In summary, the evidence of long-term neurodevelopmental harm with administration of steroids is clear with early (<8 day) administration of dexamethasone [36]. With later administration (>7 days), the data trended towards increased cerebral palsy along with a trend towards decreased mortality [37, 38].

Given these findings of increased likelihood of poor neurological outcome, current evidence is clearly against the early use of dexamethasone in the first week of life. Later use of dexamethasone should be undertaken with caution and reserved for patients with BPD in whom weaning from high ventilator settings and oxygen support is unsuccessful or their respiratory status is rapidly deteriorating.

Recent arguments have questioned the use of dexamethasone in the steroid trials and the possible role of other steroids. Betamethasone, a stereoisomer of dexamethasone, may have a differential role in preterm infants. Some have reported concern of possible direct neuronal injury and neurological side effects from the preservatives such as sulfites present in dexamathasone and potential further study of postnatal betamethasone may be warranted [40, 41]. Hydrocortisone prophylaxis for early adrenal insufficiency to prevent BPD was examined [42]. In this study, preterm infants weighing less than 1 kg and mechanically ventilated were randomized to receive placebo or hydrocortisone, 1 mg/kg/day for 12 days and then 0.5 mg/kg/day for 3 days. The study showed no significant differences in the survival rates between the two groups. However, among infants exposed to chorioamnionitis, the ones treated with hydrocortisone had significantly lower mortality and improved survival without BPD. There was no suppression of adrenal function or short-term growth but a higher rate of gastrointestinal perforation was seen in the hydrocortisone-treated group receiving indomethacin compared to the placebo group. Additional trials may be warranted in order to determine the role of low-dose hydrocortisone therapy in the prevention of BPD especially in preterm infants born to mothers with chorioamnionitis.

Inhaled steroids have also been evaluated in an effort to optimize the benefits of corticosteroids and minimize unacceptable systemic side effects. Inhaled steroids have been tried early (<2 weeks of age) to prevent BPD and later to treat established BPD [43, 44]. None of the trials demonstrated significant change on the BPD rate at 28 days or 36 weeks postmenstrual age. Multiple trials examined the effectiveness of inhaled steroids administered to ventilator dependent preterm infants after two weeks of life [45–48]. These approaches offered no advantage of aerosolized corticosteroids over systemic therapy. Aerosolized steroids did not have any significant effect on the mortality or incidence of BPD, duration of ventilatory support, or oxygen therapy. Major concerns with inhaled corticosteroids included the type of steroids, their dosages, and uncertainty regarding drug delivery. Studies have suggested that delivery of aerosolized particles is limited by the size of the particles, presence or absence of endotracheal tube to facilitate delivery, differences in delivery device (i.e., MDI versus spacer), and use of nebulizers. There is some evidence that inhaled steroids are absorbed systemically and thus carry risks similar to systemic steroids. At the time of this review, a multicenter randomized controlled clinical trial is underway in Europe (NEuroSIS) aiming to examine whether early administration of inhaled steroids in preterm infants reduces the risk of BPD and includes short-term and long-term outcomes which may answer questions regarding both efficacy as well as safety. Due to their multiple mechanisms of action, steroids continue to offer promise in the prevention and management of BPD; however, the appropriate dose, timing, and size of the glucocorticoid molecule need to be further studied in order to maximize benefit and minimize risks. These studies must include long-term pulmonary and neurodevelopmental follow-up in order to determine whether the intervention is safe and effective.

## 6. Mast Cell Stabilizer

Cromolyn, a mast cell stabilizer, is the first nonsteroidal anti-inflammatory drug used in asthmatic patients. It targets both sensitized and nonsensitized mast cells and prevents degranulation and release of histamine. Mast cell stabilizers have been shown to decrease neutrophil migration and activation thus minimizing inflammation [49]. Two trials studied the possible role of cromolyn in prevention and treatment of evolving BPD [49, 50]. Though the sample sizes were small, both studies showed no improvement in mortality, days on mechanical ventilation, or incidence of BPD. Cytokine levels were lower in the lung lavage fluid in the treatment group compared to the placebo [49]. These studies, similar to other aerosolized drug studies, did not assess drug delivery, thus failing to provide evidence for effective drug deposition. Current evidence does not support the use of cromolyn for the prevention or treatment of BPD but further studies may be warranted.

## 7. Vitamin A

Vitamin A is a retinoid essential for the normal lung growth and important in regulation of lung epithelial cell repair. It is known that preterm infants have low levels of Vitamin A at birth with low levels associated with an increased risk of chronic lung disease [51]. A Cochrane meta-analysis reviewed eight studies on the efficacy of Vitamin A supplementation in prevention of BPD. In extremely low birth weight infants, supplementation was found to decrease rates of BPD [52]. In the largest trial in the meta-analysis, infants less than 1000 grams who received vitamin A supplementation had an 8% decrease in rate of death or BPD compared to the placebo group [53]. While this meta-analysis includes eight studies, the study by Tyson et al. is clearly the largest and greatly influences the results. Enteral application of high-dose vitamin A was examined in one study that did not show any long-term positive effect [54]. The current evidence solely supports the intramuscular delivery of high-dose vitamin A to ELBW infants [53]. Neurodevelopmental outcomes at 18 to 22 months were not different in the two experimental groups; interestingly there was also no difference in respiratory outcome at 18 to 22 months [55]. Evidence supports the use of high-dose intramuscular vitamin A supplementation for the prevention of BPD in premature infants <1000 grams although there are no long-term benefits in pulmonary or neurodevelopmental outcome.

## 8. Inositol

Inositol is a phospholipid that enhances the synthesis and secretion of surfactant phospholipids thereby improving pulmonary function. A randomized controlled trial by Hallman et al. demonstrated lower oxygen and airway pressure requirements with the use of intravenous inositol; however very few patients received surfactant in this trial [56]. Within the group receiving surfactant, there was no reduction in BPD after inositol administration in this study. A Cochrane meta-analysis that included all infants who received inositol treatment showed a significant reduction in death or BPD compared to untreated controls [57]. No further studies to confirm these findings have been reported; it is possible that the positive results previously found would no longer be present in the surfactant era or that inositol administration may benefit a subpopulation of infants. Inositol is not currently recommended for prevention of BPD but further trials may be warranted in the surfactant era to confirm these preliminary findings and to study the long-term effects.

## 9. Antioxidants

**(a) Superoxide Dismutase (SOD)**

Free radicals have been implicated in the pathogenesis of BPD. Premature infants are susceptible to oxidant injury since they are relatively deficient in antioxidant enzymes while being exposed to toxic oxygen levels [58]. Preliminary animal and human studies have provided evidence for a protective action of antioxidants such as SOD in hyperoxia-induced acute and chronic lung injury [59–61]. A randomized controlled trial studied if recombinant CuZnSOD would decrease the incidence of BPD in ventilated and surfactant-treated preterm infants [62]. This trial enrolled 302 patients and showed that CuZnSOD can be given safely and is well tolerated intratracheally but found no difference in the primary outcome of BPD at 28 days of life or 36 weeks postmenstrual age. The striking result was a significant decrease in several indicators of lung disease in the treatment group over the first year of life including reduction in need for asthma medications, fewer emergency department visits, and fewer hospitalizations suggesting a delayed beneficial response at one year of age in infants <27 weeks gestation. The mechanism underlying this SOD-mediated delayed benefit is unclear but presumably involves the disruption of the pathogenic reactive oxygen species. It appears that the role of SOD in the management of BPD may warrant further study. The long-term effect of SOD in other neonatal morbidities and the effects of dosage, mode of delivery, frequency, and type of preparation of SOD need to be addressed in future trials.

**(b) N Acetyl-Cysteine (NAC)**

Glutathione is an endogenous scavenger of free radicals, which is relatively deficient in premature infants with decreasing gestational age [63]. Ahola et al. proposed use of NAC, a precursor of glutathione, to ameliorate cellular injury from free radicals [64]. Intravenous NAC was administered for the first six postnatal days in a multicenter double blind placebo-controlled trial. In a group of 391 infants weighing under 1000 g, no significant differences were found in incidence or severity of BPD between the NAC and placebo groups [64]. A follow-up study showed no significant difference in the lung function between the two groups [65]. Long-term follow-up of these infants will be required to determine potential delayed benefits.

**(c) Tocopherol (Vitamin E) and Ascorbic Acid (Vitamin C)**

Both Vitamin E and C could serve as scavengers of reactive oxygen species produced during high oxygen exposure and prevent lipid peroxidation. Randomized controlled trials have shown no evidence that vitamin E supplementation alone or in combination with vitamin C offers protection against BPD [66, 67].Although the mechanism is well established, limited success has been achieved using antioxidants and therefore their routine use is not recommended at present. Potential limiting factors include radical formation restricted to subcellular compartments, timing, dose, and delivery of the drug, or perhaps a need for multiple agents blocking different pathways of reactive oxygen species. Alternatively, as the SOD trial has shown, lack of acute benefit does not preclude delayed beneficial effects on pulmonary outcome. This underscores the importance of including long-term outcomes in the design of randomized trials of pharmacologic interventions for BPD.

## 10. Inhaled Nitric Oxide (iNO)

iNO is a selective pulmonary vasodilator that decreases pulmonary vascular resistance without affecting systemic vascular tone [68]. The rationale for the use of iNO in the prevention of BPD stems from animal and human studies supporting an anti-inflammatory role for NO and beneficial effects in lung structure and gas exchange [69–73]. Several large clinical trials with different study designs yielded variable results [74–79]. These studies included iNO given as prophylaxis to prevent BPD, as rescue therapy for severe acute respiratory failure, and as treatment for severe BPD in a variable patient population. One of the larger trials by Ballard et al. enrolled intubated infants during the second postnatal week and used a higher starting dose of iNO and demonstrated a modest reduction in BPD in the treatment group, but no difference in death [74]. While a modest reduction in composite outcome of death or BPD was found in a systematic review, there was no evidence of reduction in rates of death alone or BPD in infants treated with iNO compared to controls [68]. An individualized patient data meta-analysis of randomized trials also found that routine use of iNO for treatment of respiratory failure cannot be recommended [80]. The variable results and difficulty in interpretation of the numerous trials prompted an NIH consensus development conference which concluded that current evidence does not support use of iNO in early routine, early rescue, or later rescue regimens in the care of premature infants <34 weeks [81]. Future trials to define the optimal dose, timing, and duration of iNO therapy in prevention on BPD are warranted and are ongoing at the time of this review.

## 11. Other Potential Therapies

### 11.1. Mesenchymal Stem Cell Therapy

The therapeutic potential of stem cells is currently explored for a variety of disorders. Intrinsic qualities of mesenchymal stem cells such as their capacity to respond, migrate, and replace damaged tissue make them an attractive candidate for prevention and repair of neonatal lung injury. In animal models, bone marrow-derived mesenchymal stem cells (BMSCs) have been shown to ameliorate injury in multiple organs including heart, brain, kidney, and lung [82–85]. In neonatal rodent models of BPD, allogenic BMSCs have been shown to prevent lung injury and lung inflammation [84–86]. This protection was observed despite a very low level of BMSC engraftment in the lungs. In fact, even more profound improvement in alveolar simplification and vascular injury was seen after delivery of BMSC-conditioned media indicating that a paracrine mechanism is likely involved [84]. Further studies in animal models of BPD are needed to address whether BMSCs can provide protection by a paracrine immunomodulatory response leading to release of specific growth factors and anti-inflammatory molecules [86].

## 12. Conclusion

Well-conducted clinical trials and meta-analyses have demonstrated a lack of significant impact of several pharmacologic therapies [87]. Despite this, many pharmacologic therapies are currently practiced because of transient beneficial effects and lack of alternatives. As our understanding of the complex and multifactorial pathophysiology of BPD improves, it becomes clear that targeting individual pathways is unlikely to have a significant impact on outcome. A multidrug approach addressing several pathways simultaneously may have a more significant impact on the incidence and progression of the disease. We need to continue to work to understand the basic mechanisms of neonatal lung development, injury, and repair. In addition, targeted therapeutic approaches based on host factors and specific patient genetic and epigenetic makeup may allow better therapeutic choices. Some prediction tools have been developed based on risk factors to help provide prognostic information and facilitate identification of infants who may benefit from therapies available [88]. Continued clinical practice optimization with minimization of ventilator-induced lung injury, oxygen toxicity, and infection as well as continued optimization of nutrition should also continue to be pursued. As we gain new insight into the disease process and evaluate novel approaches, it is essential to focus not only on short-term outcomes and safety profiles but also on long-term pulmonary and neurodevelopmental outcomes.

## Figures and Tables

**Table 1:** Pharmacological interventions for prevention and management of BPD.

Class of drugs	Presumed mechanism	Main clinical responses	Major side effects	Recommended use in BPD
Caffeine	Apnea of prematurity Unknown	Reduction in days of positive pressure ventilation, reduction in BPD, lower incidence of neurodevelopmental impairment	Transient decrease in weight gain	Recommended for treatment of apnea of prematurity and prevention of BPD

Diuretics *(loop, thiazides) *	Pulmonary Edema	Decreased pulmonary edema	Electrolyte imbalance, osteopenia, ototoxicity	*Loop:* use sparingly in early evolving BPD *Thiazides:* Consider for judicious chronic use

Bronchodilators *(albuterol, ipratropium) *	Bronchospasm	Bronchodilation	Tachycardia, arrhythmias	Limit use in infants with bronchospasm and acute clinical response

Steroids *(early, moderately early, late, inhaled) *	Inflammation	Improved oxygenation, earlier extubation	Short term: hyperglycemia, hypertension, GI perforation Long term: increased risk for cerebral palsy	Last resort therapy for rapidly deteriorating pulmonary status

Mast cell stabilizer *(cromolyn) *	Inflammation	No clinical benefit	None reported	Not for routine use

Vitamin A	Impaired lung development	Small reduction in incidence of BPD	None reported	Recommended in infants <1000 grams

Inositol	Impaired lung growth	Decreased incidence of BPD	None reported	Not for routine use

Antioxidants *(SOD, NAC,Vitamin E, vitamin C) *	Oxidant injury	Delayed benefit from SOD	None reported	Not for routine use

Inhaled NO	Inflammation Oxidant stress Unknown	Possibly beneficial in reducing BPD but optimal timing, dose and duration unknown	IVH in infants <1000 g with early rescue use	Not for routine or rescue use

Modified from Baveja and Christou [10].
